# Abstracts from the 6th Infection Control Africa Network Congress 2016: Johannesburg, South Africa. 26-28 September 2016

**DOI:** 10.1186/s13756-016-0153-0

**Published:** 2017-01-10

**Authors:** Helen Wangai, Felister Kiberenge, Alex Elobu, Josephat Jombwe, Peter Ongom, Dorah Nakamwa, Alexander Aiken, Benedetta Allegranzi, Mpho Sikhosana, Wolgang Preiser, Angela Dramowski, Heather Finlayson, Tonya Esterhuizen, Jehan El Kholy, Mervat Gaber, Dina Mostafa, Fadheela Patel, Shima Abdulgader, Adebayo Shittu, Lemese Ah Tow, Mamadou Kaba, Sekai Lilian Rubayah, Helen Ngodoo Adamu, ThankGod Emmanuel Onyiche, Magdalene Nanven, Babajide Oluseyi Daini, Samuel Tolulope Ogundare, Olukemi Olugbade, Ngozi Anayochukwu-Ugwu, Olatunji Badmus, Abisola Oladimeji, Saheed Gidado, Olufemi Ajumobi, Ndadilnasiya Endie Waziri, Patrick Nguku, Adebola Olayinka, Olukemi Olugbade, Ngozi Anayochukwu-Ugwu, Abisola Oladimeji, Olufemi Ajumobi, Saheed Gidado, Ndadilnasiya Endee Waziri, Patrick Nguku, Adebola Olayinka, Mohamed Shallouf, Pedro M. D. S. Abrantes, Charlene W. J. Africa, Eltony Mugomeri, Bisrat Bekele, Charles Maibvise, Clemence Tarirai, Kenneth I. Onyedibe, Emmanuel O. Shobowale, Mark O. Okolo, Nathan Y. Shehu, Rita Pike, Shelter Nyauzame, Cynthia Chasokela, Valerie Jean Robertson, Tendai Jubenkanda, Wilson. Mashange, Junior Mutsvangwa, Gladys Dube, Rose Katumba, Alethea Mashamba, Anna Maruta, Shirish Balachandra, Kongnyu Emmanuel, Nkwan Jacob, Gideon Wiysinyuy, Buyiswa Lizzie Sithole, Boniface Hakizimana, Christiana Kallon, Barbara Burmen, James Marcomic Maragia, Mustafa Esmaio, Pedro Abrantes, Charlene Africa, Rafael Joaquim, Namaunga K. Chisompola, Elizabeth M. Streicher, Rob M. Warren, Samantha L. Sampson, Mojisola Christiana Owoseni, Anthony Okoh, Habib Yakubu, Katharine Robb, Constance Bwire, Richard Mugambe, James Michiel, Joanne McGriff, Christine Moe, Jane Ngivu, Olanrewaju Jimoh, Oluwafemi T. Ige, Zainab L. Tanko, Abdulmumin K. Mohammed, Victoria Aganabor, Busayo O. Olayinka, Abdulrasul Ibrahim, Joy O. Daniel, Adebola T. Olayinka, Joan Rout, Petra Brysiewicz, Yolanda Van Zyl, Shereen Arontjies

**Affiliations:** 1 Department of Health Services, Nyandarua County, Kenya; 2 Ministry of Health, Nairobi, Kenya; 3 Mulago Hospital, Kampala, Uganda; 4 London School of Hygiene and Tropical Medicine, London, UK; 5 World Health Organization, Geneva, Switzerland; 6 Division of Medical Virology, Stellenbosch University /NHLS, Tygerberg, South Africa; 7 Paediatric Infectious Diseases, Department of Paediatrics and Child Health, Stellenbosch University, Tygerberg, South Africa; 8 Biostatistics Unit, Centre for Evidence Based Health Care, Stellenbosch University, Tygerberg, South Africa; 9 Cairo University Hospitals (CUH), Cairo, Egypt; 10 Division of Medical Microbiology, Department of Pathology, Faculty of Health Sciences, University of Cape Town, Cape Town, South Africa; 11 Division of Medical Microbiology, Department of Pathology, Faculty of Health Sciences, University of Cape Town, Cape Town, South Africa; 12 Department of Microbiology, Obafemi Awolowo University, Ile-Ife, Nigeria; 13 Mpilo Central Hospital, Bulawayo, Zimbabwe; 14 Department of Epidemiology and Medical statistics, Faculty of Public Health, College of medicine, University of Ibadan, Oyo state, Ibadan Nigeria; 15 Department of Microbiology and Parasitology, Faculty of Veterinary Medicine, University of Maiduguri, Borno state, Maiduguri Nigeria; 16 Department of Microbiology, Faculty of Veterinary Medicine, Ahmadu Bello University, Kaduna State, Zaira Nigeria; 17 Department of Production Animal Studies, Faculty of Veterinary Science, University of Pretoria, Onderstepoort, 0110 Pretoria, South Africa; 18 Nigeria Field Epidemiology Laboratory Training Programme (NFELTP), Abuja, Nigeria; 19 Airtel Networks Limited, Abuja, Nigeria; 20 Nigeria Field Epidemiology Laboratory Training Programme (NFELTP), Abuja, Nigeria; 21 Microbial Endogenous Infections (MEnIS) Research Laboratories, Department of Medical Biosciences, University of the Western Cape, Private Bag X17, Bellville, 7535 South Africa; 22 National University of Lesotho, Roma, Lesotho; 23 University of Swaziland, Kwaluseni, Swaziland; 24 Tshwane University of Technology, Pretoria, South Africa; 25 Jos University Teaching Hospital, Jos, Nigeria; 26 Babcock University, Ikenne, Nigeria; 27 Biomedical Research and Training Institute, Harare, Zimbabwe; 28 Ministry of Health and Child Care, Harare, Zimbabwe; 29 University of Zimbabwe, Harare, Zimbabwe; 30 U.S. Centers for Disease Control and Prevention (CDC), Harare, Zimbabwe; 31 Etougebe Baptist Hospital, Yaounde, Cameroon; 32 Baptist Training School for Health Personel Banso, Yaounde, Cameroon; 33 Etouge Baptist Hospital, Yaounde, Cameroon; 34 World Health Organization, Freetown, Sierra Leone; 35 Ministry of Health and Child Welfare, Freetown, Sierra Leone; 36 Kenya Medical Research Institute Center for Global Health Research, Nairobi, Kenya; 37 Lodwar county and referral hospital, Lodwar, Kenya; 38 University of Western Cape, Cape Town, South Africa; 39 Maputo Central Hospital, Maputo, Mozambique; 40 Faculty of Medicine and Health Sciences, Department of Biomedical Sciences, Division of Molecular Biology and Human Genetics, Stellenbosch University, Cape Town, South Africa; 41 School of Medicine, Department of Basic Sciences, Copperbelt University, Ndola, Zambia; 42 University of Fort Hare, Alice, South Africa; 43 Emory University, Rollins School of Public Health, Center for Global Safe Water, Sanitation and Hygiene, Atlanta, GA USA; 44 Care International in Uganda, Kampala, Uganda; 45 Department of Disease Control and Environmental Health, Makerere University School of Public Health, Kampala, Uganda; 46 Infection Control Coordinator, Gertrude’s Children’s Hospital, Nairobi, Kenya; 47 Department of Medical Microbiology, Ahmadu Bello University /Teaching Hospital, Zaria, Kaduna State Nigeria; 48 Department of Medical Microbiology, Kaduna State University, Kaduna, Nigeria; 49 Department of Nursing services, Ahmadu Bello University /Teaching Hospital, Zaria, Kaduna State Nigeria; 50 Department of Pharmaceutics and Pharmaceutical Microbiology, Ahmadu Bello University, Zaria, Kenya; 51 Hillcrest Private Hospital, Durban, South Africa; 52 School of Nursing and Public Health College of Health Sciences University of KwaZulu-Natal, Durban, South Africa; 53 Paarl Hospital, Western Cape, Paarl, South Africa

## A1 Health facilities audit for infection prevention and control practices in Nyandarua County, Kenya

### Helen Wangai^1*^, Felister Kiberenge^2†^

#### ^1^Department of Health Services, Nyandarua County, Kenya; ^2^Ministry of Health, Nairobi, Kenya

##### **Correspondence:** Helen Wangai (hwwangai@yahoo.com)

**Background and objectives**

Infection prevention and control is a set of practices, protocols, and procedures that are put in place to prevent infections that are associated with health care service provision settings. It is an element of quality of care and safety in health care service delivery; health worker occupational health and safety practices; medical waste management; and is also concerned with clinical and public health surveillance and action. Healthcare facilities are ideal settings for the transmission of infections to patients (who are more susceptible), healthcare workers, their families and communities. Healthcare associated infections lead to prolonged hospital stay, increased cost of care and death. Therefore, the objective of this study was to assess infection prevention and control practices in various types and levels of health facilities in Nyandarua County, Kenya.

**Methodology**

A cross-sectional descriptive study was conducted in 47 health facilities that were sampled from a total of 153, using cluster sampling technique. The facilities were drawn from public, private and faith based organizations distributed across 5 sub-counties of Nyandarua County, Kenya. Data collection method was purely quantitative using a structured questionnaire. Descriptive analysis was done using SPSS version 17.

**Results**

42/47 of the facilities were observing safe injection practices. Only 8/26 of the public health facilities had all their workers immunized against hepatitis B despite procurement, supply and distribution of adequate vaccine doses for the entire health workforce. Poor medical waste management practices where 37/47, 15/47 and 28/47 were segregating waste, had colour coded bins and had functional incinerators respectively.

Only 28/47of the sampled facilities were decontaminating patient reusable equipment/instruments appropriately. Electricity, gas, charcoal and firewood were fuels used for autoclaving. About 33/47 and 30/47 of the sampled facilities had running water and soap/hand disinfectant respectively.

**Conclusions**

The findings revealed several gaps in the implementation of the national IPC policy especially in healthcare worker occupational health and safety, reprocessing of patient reusable equipment/instruments, medical waste management and hand hygiene practices. These findings will assist the department of health in designing interventions for strengthening and improving IPC practices, to mobilize and allocate resources for IPC activities, improve infrastructure and supplies in line with the strategic priority 5 of the national strategic plan for IPC for health care services in Kenya. The study findings will also provide county specific baseline data that will help in: measuring future performances, in research and add to the existing knowledge of other scholars on infection prevention and control.

**Acknowledgments**

“This work is based on the research supported by the National Research Foundation”

*Oral presentation prize: Silver ICAN medal*

## A2 Prevention of surgical site infections in a low resource setting; experience in Mulago Hospital, Kampala, Uganda

### Alex Elobu^1^, Josephat Jombwe^1^, Peter Ongom^1^, Dorah Nakamwa^1^, Alexander Aiken^2^, Benedetta Allegranzi^3^

#### ^1^Mulago Hospital, Kampala, Uganda; ^2^London School of Hygiene and Tropical Medicine, London, UK; ^3^World Health Organization, Geneva, Switzerland^3^

##### **Correspondence:** Alex Elobu (elobuemmy@gmail.com)

**Background**

In low-income settings, surgical site infections (SSI) represents the commonest form of healthcare-associated infection. Although largely preventable, SSIs continue to have high prevalence and mortality and there is also an apparent deficiency in research relating to this topic in Africa. This study sought to evaluate an intervention package for preventing SSI in a low-resource setting.

**Methods**

This was a before-and-after interventional study conducted at Mulago Hospital, Uganda between January 2014 and June 2015. This was part of a larger multi-Centre study involving five African hospitals. After an initial baseline SSI surveillance period, we introduced the intervention which included the following process measures: 1) preoperative patient bathing, 2) minimization of hair removal, 3 + 4) appropriate surgeons hand and patient skin preparation, 5) administration of peri-operative prophylactic antibiotics and 6) maintenance of discipline in the operating room. The intervention also promoted efforts to address the wider culture of patient and facilitated inter-hospital communication. The outcomes of interest were SSI incidence and 30-day mortality.

**Results**

We recruited a total of 841 patients (493 in baseline phase; 348 intervention phase) between February 2014 and May 2015. This included a wide range of both elective and emergency procedures, predominantly abdominal and gastrointestinal surgery. The following changes in process measures were recorded between baseline and intervention phases: proportion with preoperative bath 72% to 79% (p = 0.016), proportion with hair not removed 71.5% to 90.5% (p < 0.001), quality of surgeon’s hand preparation “low” 10.6% to 2.6% and “high” 54.1% to 62.9% (p < 0.001), use of alcohol based skin preparation from 0.8% to 73.9% (p < 0.001). Discipline in the operating room improved with reduction in average number of persons entering the room from 4.1 to 2.9 and door openings from 7.8 to 4.7 times. There was a significant reduction of 57% in SSI rate from 14% to 6% (p < 0.001) but no significant change in mortality 3.3% to 1.4% (p = 0.098).

Important challenges to the implementation of the intervention phase included the death of the lead investigator, inertia for behavior change among staff and frequent rotation of staff. Major facilitating factors included support from hospital leadership and communication with other participating study sites.

**Conclusion**

Our intervention bundle led to a 57% reduction in SSI. This otherwise inexpensive intervention package can be effective in reducing SSI occurrence and should be adopted in other low resource hospital settings.

**Acknowledgments**

“This work is based on the research supported by the National Research Foundation”

*Oral presentation prize: Gold ICAN medal*

## A3 Seroprevalence of measles, rubella, varicella-zoster, hepatitis A and hepatitis B virus antibodies in first year medical students in the Western Cape, South Africa

### Mpho Sikhosana^1^, Wolgang Preiser^1^, Angela Dramowski^2^, Heather Finlayson^2^, Tonya Esterhuizen^3^

#### ^1^Division of Medical Virology, Stellenbosch University /NHLS, Tygerberg, South Africa; ^2^Paediatric Infectious Diseases, Department of Paediatrics and Child Health, Stellenbosch University, Tygerberg, South Africa; ^3^Biostatistics Unit, Centre for Evidence Based Health Care, Stellenbosch University, Tygerberg, South Africa

##### **Correspondence:** Mpho Sikhosana (lsikhosana@gmail.com)

**Background & objectives**

Medical students are exposed to blood- & airborne infections during their training. Nonimmune students acquiring viral infections may transmit organisms to colleagues & vulnerable patients, potentially triggering nosocomial outbreaks. Many viral infections are vaccine preventable, and determining students’ immune status and providing appropriate vaccination before patient contact would be beneficial. The objectives of this study, the first of its kind in our setting, were to determine the seroprevalence of antibodies to 5 vaccine preventable viral diseases (VPVDs: measles, rubella, varicella-zoster [VZV], hepatitis A [HAV] and B [HBV]) among 1st year medical students in a medical school, and to determine the reliability of self-reported vaccination history. Findings are envisioned to inform recommendations for the vaccination of healthcare students at the university.

**Methods**

This was a cross-sectional study undertaken in 2015 at the Stellenbosch Faculty of Medicine and Health Sciences, Tygerberg Campus. 290 first year medical students were approached and informed about the study. Information sessions explaining the rationale of the study were conducted, after which willing participants were to complete a questionnaire regarding their demographic information and vaccination history, and provide a blood sample for the measurement of IgG antibodies against the VPVDs screened. Proof of previous vaccination was not sought from the participants. Questionnaires and serology data were anonymized. Unique study numbers, with which to look up their results on the university student website, were given to each participant after recruitment. No further steps were taken regarding nonimmune participants, but students were advised to take appropriate measures if found to be non-immune. SPSS was used for data analysis.

**Results**

210 participants were enrolled (72.4% participation rate). 140 (66.7%) were female, 70 (33.3%) were males, and the mean age was 19.3 years (range 17-41). All except 3 participants were born in South Africa. Seroprevalence rates found were 73.8%, 84.3%, 81%, 83.8%, 81.4% for HAV, HBV, measles, rubella & VZV respectively. 65.4% of participants received a HBV booster as per university requirements, which could have affected the HBV seroprevalence rate obtained. There was no statististical significance with regards to seropositivity to any of the VPVDs between the group of participants who gave a positive previous childhood vaccination history and those who did not.

**Conclusions**

Though reasonable levels of immunity were found in the 1st year medical students, immunity gaps still exist and self-reporting history was found not to be predictive of immune-status. Implementation of screening and vaccine booster programmes would address such gaps.

## A4 Healthcare-associated infections (HAI) in Cairo University Hospitals (CUH): a success story of surveillance in a resource- limited country

### Jehan El Kholy, Mervat Gaber, Dina Mostafa

#### Cairo University Hospitals (CUH), Cairo, Egypt

##### **Correspondence:** Jehan El Kholy (jehanelkholy12@gamil.com)

**Background**

Health care-associated infections are the most frequent adverse event in health-care delivery worldwide. Limited data are available from low- and middle-income countries. Most countries lack surveillance systems for health care-associated infections. We aimed to describe the results of the surveillance system we followed from 1 September 2014 till 31 March 2016 in Cairo University Hospitals (CUH) a 5200 bed- tertiary hospital.

**Methods**

Standardized surveillance system was conducted in all intensive care units (ICU) of CUH from 1 September 2014 to 31 March 2016. Surveillance was active prospective and focused on ICU patients; a vulnerable patient population at increased risk of HAI and AMR due to severity of illness, high exposure to invasive procedures and devices, and high use of broad spectrum antibiotics. HAI definitions used were the same 2008 NHSN case definitions. The involvement and training of IC Team, data entry person, Microbiology laboratory performing full bacterial identification, antimicrobial susceptibility testing, and culturing all types of specimens, IPC link nurses in ICUs to monitor and report infections to the IPC team were essential. Data were collected with surveillance officers and analyzed. Device-days were used to calculate incidence of device-associated infections and patient-days to calculate incidence of HAIs that were not device-related. Antimicrobial susceptibility testing was performed using CLSI guidelines.

**Results**

Thirty- eight ICUs including medical, surgical, stroke units, cardiac, cardio-surgical, obstetric, pediatric, neonatal and burn units contributed to 94877 patient- days and 1272 HAIs. Of these 224 (18%) are ICU acquired, 111 (9%) Ward acquired, 808 (63%) Infections present on ICU admission and 129 (10%) SSI. Of the infections BSI represented 43% (with 70% CLABSI), UTI represented 27% (with 97% CAUTI) and pneumonia represented 39% (with VAP 80%). The incidence of HAI was 2.4/ 1000 patient- days, VAP was 2.5/ 1000 ventilator days, CLABSI was 1.2/ 1000 central line days and CAUTI was 1.2/ 1000 urinary catheter days.

Culture of microorganisms showed that gram negative pathogens constituted 71.4% of the total pathogens, mainly *Klebsiella* spp. constituted (28.6%), *Acinetobacter spp*. (16.6%), and *Pseudomonas spp.* (9.4%). Most of *Acinetobacter* and *E. coli* isolates were multi-drug resistant; 83.7% and 82.7%, respectively.

**Conclusion**

Implementing a standardized surveillance system in a resource-limited country is possible. Having a continuous and sustainable surveillance system is a success. Surveillance is fundamental to have benchmark of infections, to plan for prevention strategies, to record the antimicrobial resistance pattern and to plan for an antimicrobial stewardship program.

## A5 Prevalence of Clostridium difficile infection in Africa: a systematic review and meta-analysis

### Fadheela Patel^1^, Shima Abdulgader^2^, Adebayo Shittu^3^**,** Lemese Ah Tow^1^, Mamadou Kaba^1^

#### ^1^Division of Medical Microbiology, Department of Pathology, Faculty of Health Sciences, University of Cape Town, Cape Town, South Africa; ^2^Division of Medical Microbiology, Department of Pathology, Faculty of Health Sciences, University of Cape Town, Cape Town, South Africa; ^3^Department of Microbiology, Obafemi Awolowo University, Ile-Ife, Nigeria

##### **Correspondence:** Fadheela Patel (mamadou.kaba@hotmail.com)

**Background and objectives**

Clostridium difficile infection (CDI) is one of the most common causes of healthcare-associated diarrhoea worldwide, with substantial morbidity and mortality rates. Treatment and prevention of CDI is complex because of the emergence of antibiotic resistance. This systematic review determined the prevalence and risk factors of CDI in the African population.

**Methods**

Eligible articles (in English and French) were searched in various databases (Scopus, PubMed, Web of Science and EBSCOhost) throughout April 2016, and analysed by three independent reviewers. Meta-analysis for proportion was achieved by determining faecal positivity rates for Clostridium difficile (CD) as reported in the eligible studies

**Results**

We identified 19 articles of which 42% (8/19) were reported on adults, 11% (2/19) on children, 26% (5/19) on both children and adults, while the remaining did not report on the age groups. Most of the studies (80%, 16/19) were cross-sectional. Six of the 19 articles (32%) included in this review were conducted in South Africa. Most of the CDI were diagnosed using phenotypic tests (74%, 14/19). Other studies employed genotypic identification methods (10.5%, 2/19) and 16% (3/19) of the studies applied both phenotypic and genotypic methods. Two studies included data on asymptomatic participants. The combined CDI prevalence (based on 18 studies) was 14% (95% CI: 6%-25%). CDI was not detected in studies conducted in Kenya and Zambia. An equal number of studies (n = 6) reported on either community-associated (CA) or hospital-associated (HA) CDI. Moreover, five of the 19 studies (26.3%) investigated a combination of HA and CA infections. The pooled prevalence for CA-CDI and HA-CDI was 13% (95% CI: 8%-36%) and 15% (95% CI: 12%-17%), respectively. Risk factors associated with CDI based on multivariate analysis as reported by only two studies were antibiotic usage (OR, 2.9; 95% CI: 1.6-5.1) and duration of hospitalization (OR, 23.6; 95% CI: 1.2-4.53). Three articles reported on antibiotic susceptibility testing. In one study conducted in Zimbabwe, the CD isolates were resistant to clindamycin, gentamicin, cefotaxime, and ciprofloxacin. Moreover, 17.4%, 26.1%, 43.5% and 70% of the isolates exhibited resistance to tetracycline, ampicillin, erythromycin and cotrimoxazole, respectively. In two other studies, all the CD isolates were susceptible to metronidazole and vancomycin.

**Conclusion**

This review highlights the lack of data on CD carriage and/or infection in Africa, nevertheless the CD prevalence (14%) in diarrheic stool of patients in Africa is not negligible. Therefore, there is a need for more surveillance studies on CDI in Africa.

## A6 Health worker TB screening uptake at Mpilo Central Hospital, Bulawayo, Zimbabwe

### Sekai Lilian Rubayah (slrubayah@hotmail.com)

#### Mpilo Central Hospital, Bulawayo, Zimbabwe

**Background**

Mpilo Central Hospital has an establishment of 1670 workers. About 160 000 patients are seen at the hospital each year. Most clinical areas are usually crowded with limited ventilation due to design and non-functional mechanical ventilation, putting health workers at continuous risk of acquiring airborne infections like tuberculosis (TB). Zimbabwe is a TB endemic country and an average 70 TB cases are confirmed every month at Mpilo Central Hospital. Occupational health is a key component of standard precautions of which the employer has a major responsibility. Annual TB screening for health workers has been made a National Policy in Zimbabwe, however strategies to operationalize this at facility level have not been established. The Mpilo Hospital Infection Prevention and Control (IPC) department was tasked to put in place a system of screening health workers for TB at the hospital. We developed and tested a TB screening strategy, and here we describe the outcomes.

**Methods**

The TB screening exercise was voluntary and conducted over a period of one month from 15 October to 15 November 2015. The National TB screening tool was used to identify healthcare workers who could be having TB. The screening process was confidential and was done at departmental level by the responsible doctor/nurse-in-charge. Hospital executive, matrons and doctors were screened in the IPC department of the hospital. A TB presumptive register for staff was developed and the presumptive TB cases were referred for X-ray. Data analysis was done in the IPC department.

**Results**

A total of 751/1650 (45.5%) healthcare workers were screened. Of these 310 nurses, 10 doctors, 210 support staff (laboratory, x-ray, physiotherapists, receptionists, etc.) and 221 cleaners and general hands. Fifty-six presumptive TB cases were identified and two nurses, one cleaner and one laundry worker were confirmed to have TB. One of the two nurses died. Stigma, inadequate knowledge and fear of losing employment were among the reasons for poor uptake of the TB screening opportunity amongst health workers at Mpilo Hospital.

**Conclusion**

There is need to train health workers at Mpilo Central Hospital on the importance of occupational health so that they are at the forefront when it comes to issues to do with their well-being such as uptake of TB screening opportunities.

**Acknowledgments**

“This work is based on the research supported by the National Research Foundation”

*Best Poster Prize – Silver ICAN medal*

## A7 Community knowledge, perception and practice regarding Ebola prevention in FCT, Nigeria

### Helen Ngodoo Adamu^1^, ThankGod Emmanuel Onyiche^2^**,** Magdalene Nanven^3^, Babajide Oluseyi Daini^1^, Samuel Tolulope Ogundare^4^

#### ^1^Department of Epidemiology and Medical statistics, Faculty of Public Health, College of medicine, University of Ibadan, Oyo state, Ibadan, Nigeria; ^2^Department of Microbiology and Parasitology, Faculty of Veterinary Medicine, University of Maiduguri, Borno state, Maiduguri, Nigeria; ^3^Department of Microbiology, Faculty of Veterinary Medicine, Ahmadu Bello University, Kaduna State, Zaira, Nigeria; ^4^Department of Production Animal Studies, Faculty of Veterinary Science, University of Pretoria, Onderstepoort, 0110, Pretoria, South Africa

##### **Correspondence:** Helen Ngodoo Adamu (adamuhelen66@yahoo.com)

**Background and objectives**

Nigeria, a West African country successfully controlled an outbreak of Ebola virus disease in August 2015. Documented report of Ebola outbreaks in affected communities was associated with rumors, fear and poor perception of the disease and stigmatization of Ebola survivors in their host communities. The level of knowledge of community members to Ebola disease can have an influence on making informed decision regarding their risk behavioral practices and attitude to survivors.

**Methods**

This cross-sectional study used multistage sampling techniques to select a total of 1190 respondents from two area councils in FCT, Abuja (Bwari and Abuja municipal area council). A pretested interviewer administered questionnaire which included 10-point knowledge score on Ebola, 10-point scale on attitude of community members to survivors of Ebola, 6-point scale on practice and 5-point scale on perception was used to collect information from respondents. Knowledge score of ≥ 5, attitude score of ≥ 5, Practice score of ≥ 3, and perception score of ≥ 5 was considered as good respectively. Data analysis was done using descriptive analysis, chi-square and logistic regression at a significance level of 0.05.

**Results**

Mean age of respondents was 29.61 ± 7.51 years, 51.8% females, 43% dependents and retirees. 97.3% were aware of Ebola, media (81.7%) was their first source of information on EVD and the most trusted (80.4%). Majority (82.1%) of the respondents interviewed had good knowledge of Ebola. Eighty-three percent (83.3%) of respondents showed poor attitude to survivors of Ebola while thirty-eight percent (38%) of respondents had poor practice towards EVD prevention. About 96.3% of respondents had a high perception score.

**Conclusion**

Although Knowledge towards Ebola was high, media campaigns needs to be targeted in areas of attitude so as to avoid stigmatization and its attendant consequences by host communities towards this vulnerable person. Improved behavioral practices like regular washing of hands with soap and water should be encouraged towards Ebola virus disease prevention.

## A8 Ebola Virus Disease outbreak: epidemiologic profiles and outcomes among health workers and non-health workers, a retrospective study - Nigeria 2015

### Olukemi Olugbade^1^, Ngozi Anayochukwu-Ugwu^1^, Olatunji Badmus^2^, Abisola Oladimeji^1^, Saheed Gidado^1^, Olufemi Ajumobi^1^**,** Ndadilnasiya Endie Waziri^1^, Patrick Nguku^1^**,** Adebola Olayinka^1^

#### ^1^Nigeria Field Epidemiology Laboratory Training Programme (NFELTP), Abuja, Nigeria; ^2^Airtel Networks Limited, Abuja, Nigeria

##### **Correspondence:** Olatunji Badmus (Olatunji.Badmus@ng.airtel.com)

**Introduction**

Ebola Virus Disease (EVD) is a highly infectious viral hemorrhagic disease, with significant potential for nosocomial spread. Between July and September 2014, an outbreak of EVD occurred in two densely populated urban cities in Nigeria. We described epidemiologic profiles and outcomes of cases in this outbreak.

**Methods**

A retrospective review of clinical data on EVD cases managed in Lagos and Port-Harcourt, Nigeria was conducted. A case of EVD was defined as laboratory confirmation of Ebola virus infection in persons with fever > 38 °C or EVD compatible symptoms, or history of contact with a confirmed EVD case 21 days prior to diagnosis. The cases were categorized into two: health-workers and non-health workers. Risk factors for contracting infection identified and compared between the categories using chi-square tests.

**Results**

There were 20 cases, 11 (55%) were health workers. Compared to non-health workers, a higher proportion of health workers were aged 25 - 34 years (58% vs 42%), were females (64% vs 36%), were married (64% vs 36%), had physical contact with index case (53% vs 47%), had contact with body fluids of index case (60% vs 40%) and had greater than five days’ mean interval between onset of fever and isolation (67% vs 37%). Compared to health workers, non-health workers had a shorter hospitalization period as well as a shorter recovery period (Odds ratio (OR) = 0.08, 95% Confidence Interval (CI): 0.01- 0.95). Case Fatality Rate was higher in health workers (45%), than Non-health-workers (33%); however, there was no marked difference in Survival, which was similar in both categories, based on their occupation (O.R: - 0.6, C.I: - 0.10- 3.72).

**Conclusions**

Health workers are at higher risk of morbidity compared with non-Health workers during outbreaks of infectious diseases. Observance of standard precaution, adherence to stringent infection, prevention and control practices and timely case management will limit spread of nosocomial infections in health workers.

Keywords: Nosocomial transmission, Survival, Ebola Virus Disease, Outbreak, Nigeria

## A9 Ebola Virus Disease in healthcare settings; implications for infection control - Nigeria 2014

### Olukemi Olugbade, Ngozi Anayochukwu-Ugwu, Abisola Oladimeji, Olufemi Ajumobi, Saheed Gidado, Ndadilnasiya Endee Waziri, Patrick Nguku, Adebola Olayinka

#### Nigeria Field Epidemiology Laboratory Training Programme (NFELTP), Abuja, Nigeria

##### **Correspondence:** Olukemi Olugbade (titilope_nc@yahoo.com)

**Introduction**

In July 2014, an outbreak of Ebola Virus Disease occurred in Nigeria following its importation by an air traveler. Probable risk factors for EVD were assessed in the health facility where the index case was managed; in order to guide infection control practices in future outbreaks.

**Methods**

A health facility EVD case was any clinical staff that cared for, or had close contact with the index case and subsequently developed the disease. We conducted a retrospective review of clinical history, sociodemographic characteristics, job cadre, and type of contact with index case, of infected health workers. We tested association between potential risk factors and developing EVD in these persons, to obtain risk ratios.

**Results**

A total of 11 health workers were infected and five died (CFR 45.5%). Of those infected, 6 (54.5%) were females, and 4 (36.4%), were nurses. Also 8 health workers (72.8%) had direct physical contact and 3 health workers (27.2%) had contact with body fluid of index case. Of 5 deaths, 3 (60%) were females and 3 (60%) were doctors

The associated risk factor for contracting EVD in this outbreak was age >40 years (RR: 4.2 95% CI: 1.14-58.22). There was no association with other potential risk factors considered (p values >0.05). The study showed that exposed health workers contracted EVD irrespective of sex, marital status, job cadre and type of contact with index case - either direct physical contact or contact with body fluids.

**Conclusions**

Failure to adhere to strict infection control practices is associated with transmission of EVD among health workers. Adherence to strict infection control practices is mandatory to prevent nosocomial transmission of EVD in health care settings.

Keywords: Infection control, Health workers, Ebola Virus Disease, Outbreak, Nigeria

## A10 Carbapenem resistance expressed by Gram-negative bacilli isolated from a cohort of Libyan patients

### Mohamed Shallouf, Pedro MDS Abrantes, Charlene WJ Africa

#### Microbial Endogenous Infections (MEnIS) Research Laboratories, Department of Medical Biosciences, University of the Western Cape, Private Bag X17, Bellville 7535, South Africa

##### **Correspondence:** Mohamed Shallouf (mohamed.shallouf@gmail.com)

**Background and objectives**

Carbapenem-resistant *Enterobacteriaceae* (CRE) and other Gram-negative bacteria are among the most common pathogens responsible for both community and hospital acquired infection. The global spread of cephalosporinases in *Enterobacteriaceae* has led to the increased use of carbapenems resulting in the emergence and rapid spread of CRE. This has become an alarming public health concern, yet the condition in Libya remains unclear. The aim of this study was to obtain a better understanding of CRE strains prevalent in Libyan patients by investigating their phenotypic characteristics and antibiograms.

**Methods**

Gram-negative bacterial species were collected from Misrata Central Hospital, Misrata Cancer Centre and Privet Pathology Laboratories. Clinical samples and swabs were obtained from hospitalised and non-hospitalised patients and from mechanical ventilation and suction machines. Patients who had received antibiotic therapy for at least three days prior to the study were excluded. The identification and characterization of the isolated species were achieved using the growth characteristics on MacConkey and blood agar, spot tests and API 20E or API 20NE biochemical testing systems. Screening for carbapenem resistance was performed using the disk diffusion method with carbapenem 10 μg and cephalosporin 30 μg disks and minimum inhibitory concentrations (MIC) determined using the Sensititre Gram-negative Xtra plate format (GNX2F). All strains demonstrating resistance or reduced susceptibility to one of the four carbapenems were subjected to carbapenememase activity detection using the RAPIDEC CARBA NP test, Modified Hodge test and carbapenem inactivation methods.

**Results**

A total of one hundred and forty isolates representing fourteen bacterial species were isolated from 140 non-duplicated specimens. Clinical specimens included urine samples (96/140, 68.57%), sputum (15/140, 10.71%), surgical wound swabs (18/140, 12.85%), foot swabs from diabetes mellitus (DM) patients (6/140, 4.29%), ear swabs (3/140, 2.14%) and wound swabs (2/140, 1.43%). Thirty-four (24.29%) isolates demonstrated resistance to at least one of the four carbapenems with *Klebsiella pneumoniae* representing 73.53% (25 isolates) of all carbapenem resistant species, followed by 8.82% for *Pseudomonas aeruginosa* (3 isolates), 5.88% for both *Proteus mirabilis* (2 isolates) and *Escherichia coli* (2 isolates) and 2.94% for both *Citrobacter koseri* (1 isolate) and *Rahnella aquatilis* (1 isolate). The other isolates were either susceptible or cephalosporinase producers.

**Conclusion**

This study has revealed the high rate of carbapenem resistance amongst Libyan patients and emphasizes the crucial need for accurate screening, identification and susceptibility testing to prevent further spread of nosocomial and community acquired resistance. This may be achieved through the establishment of antibiotic stewardship programmes along with firm infection control practices.

**Acknowledgments**

“This work is based on the research supported by the National Research Foundation”

*Best poster prize: Gold ICAN medal*

## A11 High risk occupational groups and covariates for tuberculosis treatment outcomes in Lesotho

### Eltony Mugomeri^1^, Bisrat Bekele^1^, Charles Maibvise^2^, Clemence Tarirai^3^

#### ^1^National University of Lesotho, Roma, Lesotho; ^2^University of Swaziland, Kwaluseni, Swaziland; ^3^Tshwane University of Technology, Pretoria, South Africa

##### **Correspondence:** Eltony Mugomeri (emugomeri@yahoo.com)

**Background and objectives**

Tuberculosis (TB) is a serious global public health problem. To reduce the incidence of TB, particularly in high TB burden settings in Africa, the WHO, in 2010, launched the Three I’s programme comprising intensified case finding, isoniazid preventive therapy, and infection control (Kranzer et al., 2010). Lesotho, a sub-Saharan developing country with the second highest HIV burden (GoL, 2015), and the fourth highest TB prevalence (WHO, 2014), globally, launched the Three I’s programme in 2013(GoL, 2013). However, data on TB treatment outcomes and the associated covariates are scarce in the country. Therefore, this study assessed the TB treatment outcomes and the associated covariates, and identified high risk groups that need infection prevention and control (IPC) interventions in Lesotho.

**Methods**

This was a retrospective cohort review of patient records across the baseline period (2010-2012) and after the launch of the Three I’s intervention programme (2013-2015). Patients’ records at Senkatana, the largest HIV and TB clinic in Maseru, Lesotho, were reviewed between March and April 2016 based on systematic random sampling. Variables associated with unsuccessful TB treatment outcomes were determined using logistic regression.

**Results**

In total, 812 (38.1%) out of 2,132 patients’ records were included in the final analysis. About 55.2% (n = 812) were males, 83.0% were HIV-positive and 84.7% were new TB cases. Notably, factory workers (28.4%, n = 197), miners/ ex-miners (23.4%), taxi drivers (14.7%), security services personnel (8.1%) and health workers (4.6%) were the most predominant occupations among the study participants, while the least common occupations included teachers (3.0%), funeral parlor attendants (0.51%) and dry cleaning attendants (0.51%).Overall, 28.9% (n = 812) cases comprising 89 (11.0%, n = 235) defaults, 83 (10.2%) treatment failures, 49 (6.0%) deaths and 13 (1.6%) transfer-out cases, had unsuccessful treatment outcomes. Male gender (Odds ratio (OR): 1.4; 95% Confidence Interval (C.I): 1.0–1.8; P = .046), extra-pulmonary TB (OR: 3.5; 95% C.I: 2.7–4.6; P = .002) and treatment observation by a community health worker (OR: 6.2; 95% C.I: 4.0–10.0; P = .001) were significant covariates associated with unsuccessful treatment outcomes in multivariate analysis. Overall, treatment outcomes did not differ significantly (P = .636) before and after the launch of the Three I’s programme.

**Conclusion**

The implementation of the Three I’s programme in Lesotho needs to be strengthened. The covariates identified in this study are useful for policy review, while the high risk groups identified in this study highlights the need to unravel contextual underpinnings affecting IPC efforts in Lesotho.

## A12 Large focus of community acquired Extended Spectrum Beta Lactamase (ESBL) producing *Escherichia coli* in North Central Nigeria and associated risk factors

### Kenneth I Onyedibe^1^, Emmanuel O Shobowale^2^, Mark O Okolo^1^**,** Nathan Y Shehu^1^

#### ^1^Jos University Teaching Hospital, Jos, Nigeria; ^2^Babcock University, Ikenne, Nigeria

##### **Correspondence:** Kenneth I Onyedibe (kenonyedibe@yahoo.com)

**Background and objectives**

We determined the prevalence of community acquired Extended Spectrum Beta Lactamase (ESBL) *Escherichia coli* and associated risk factors in North Central, Nigeria to create awareness and prevent further emergence.

**Methods**

This was a cross sectional study carried out in Jos University Teaching Hospital. A total of 220 consenting patients with clinical isolates of E. coli were evaluated. The *E coli* isolates were tested for production of ESBL by the double disc synergy test. Control strains were used as appropriate. Data were collated and analyzed using Epi Info version 3.5.2.

**Results**

Of the 220 *E. coli* isolates, 123 (56%) were from outpatients (community acquired) out of which 16 (13%) were ESBL positive. Twenty-five (25.8%) of the 97 *E coli* isolates from inpatients (either community or hospital acquired) were also ESBL positive. Female patients were 122 (55.5%). Mean age was 36.7 ± 21.6 years. Most of the isolates were from urine (179 (81.4%)). Sixteen (7.3%) were from blood cultures, 14 (6.4%) from swabs and 11 (5.0%) from various aspirates. Multiple logistic regression analyses of the risk factors showed that only prior use of a third generation cephalosporin was statistically significant by both odds ratio (17.6) and P value (0.045).

**Conclusion**

There is a large proportion of community acquired ESBL producing *E. coli* in Northern Nigeria and prior use of a third generation cephalosporin independently contribute to emergence of this resistant phenotype. Government policies restricting antibiotic availability and use and institutional antibiotic stewardship programs are highly recommended.

## A13 Renovations for reducing airborne infections in OPD/OI Clinics - the Zimbabwe Infection Prevention Control Program (ZIPCOP)

### Rita Pike^1^, Shelter Nyauzame^1^, Cynthia Chasokela^2^, Valerie Jean Robertson^3^, Tendai Jubenkanda^1^, Wilson. Mashange^1^, Junior Mutsvangwa^1^, Gladys Dube^1^, Rose Katumba^1^, Alethea Mashamba^1^, Anna. Maruta^1^, Shirish Balachandra^4^

#### ^1^Biomedical Research and Training Institute, Harare, Zimbabwe; ^2^Ministry of Health and Child Care, Harare, Zimbabwe; ^3^University of Zimbabwe, Harare, Zimbabwe; ^4^U.S. Centers for Disease Control and Prevention (CDC), Harare, Zimbabwe

##### **Correspondence:** Rita Pike (rpike@brti.co.zw)

**Background & challenges**

A report published in 2010 assessing TB infection control across 33 health facilities in Zimbabwe showed poor patient flow in patient waiting areas and erratic water supply in critical wards. The use of waiting area space in OPD and OI for administration of HIV and TB patients care resulted in overcrowding and inadequate ventilation, increasing the risk of transmission of respiratory infections. The Zimbabwe Infection Prevention and Control Project (ZIPCOP), established in 2011 and funded by CDC through PEPFAR, aims to strengthen Zimbabwe’s national infection prevention and control (IPC) program; among its activities were minor structural renovations to address these issues.

**Methods/Activity**

The ZIPCOP team identified health facilities requiring renovations using a Renovations Eligibility Assessment tool to investigate patient waiting areas, consultation rooms, water supplies, laundry, hand washing and waste management facilities. The health facilities selected were approved by the MoHCC, and renovations were completed through the Ministry of Public Construction & National Housing, while progress was monitored by the ZIPCOP team. Each facility participated in the national IPC training programme which included site support visits. An impact assessment tool was used to determine the successful completion of the renovations.

**Results**

Ten health facilities were renovated between April 2012 and March 2016, at a cost ranging from $2000 to $3000 per facility. The renovations included 7 waiting area sheds (made of steel structure with IBR sheeting for the roof and a capacity for 25 to 400 patients) and benches, a ward for patients with multi-drug resistant TB, 8 water storage tanks, installation of precast concrete laundry tubs and linen work counters, and installation of hand washing basins and elbow taps in consulting rooms. The impact assessment tool showed improvements in IPC practices including the decongestion of patient waiting areas, improved ventilation and handwashing practices, triaging of TB patients, and improved laundry management.

**Conclusion**

Low cost interventions for renovations can contribute to meaningful infection prevention and control outcomes; this includes the routine observation of overcrowding and patient flow, and maintenance & repair of health facilities OPD/OI clinics.

## A14 Promoting safe injection practices in injections and treatment rooms of the Cameroon Baptist Convention Health Services (CBCHS): case study of Etougebe Baptist Hospital Yaounde (EBHY)

### Kongnyu Emmanuel^1^, Nkwan Jacob^2^, Gideon Wiysinyuy^3^

#### ^1^Etougebe Baptist Hospital, Yaounde, Cameroon; ^2^Baptist Training School for Health Personel Banso, Yaounde, Cameroon; ^3^Etouge Baptist Hospital , Yaounde, Cameroon

##### **Correspondence:** Kongnyu Emmanuel (kongnyu@gmail.com)

**Background**

Improper use of syringes, needles, and medication vials has resulted in patient-to-patient transmission of bloodborne pathogens (Gina Pugliese et al., 2010). In low income settings, it is estimated that up to 160 000 human immunodeficiency virus (HIV), 4.7 million hepatitis C and 16 million hepatitis B infections each year are attributable to these practices (Michelle Kermode, 2004). The CBCHS is a Faith Based Organization (FBO) noted for significant improvements in health care in Cameroon, especially in infection prevention. EBHY is one of seven CBCHS hospitals that administers an estimated 40200 injections annually with 65% considered unsafe. This facility was chosen for this study following a survey conducted in 2013 in which unsafe injection practices were observed to be more prevalent in this unit than other CBCHS facilities.

**Method**

Fourteen treatment room nurses (one BSN, two state registered nurses and eleven assistant nurses) who have as their primary role to administer injections, perform circumcisions and administer other treatments were trained in April 2014 on injection safety modules: vials/infusion bags and administration sets, needles/syringes, aseptic technique, hand hygiene and sharp containers for 17 hours. Supervision was intensified and regular weekly reminders on injection safety tips. The World Health Organization (WHO) monitoring checklist was adopted and used weekly for monitoring of injection practices from June to December 2015. The results were rated as satisfactory, needs improvement and unsatisfactory.

**Results**

The appropriate single use of vials improved from an estimated 37% in 2014 to 94.3%, (satisfactory), the use of IV bags to reconstitute medication reduced by 97%. Smaller volumes were used only during stock out of diluents (satisfactory), but a new syringe/needle was used each time to access it. Single use of needles and syringes improved from 68% to 92% (satisfactory), aseptic technique from 33% to 40% (needs improvement). The appropriate use of disposable sharps containers was rated at 95% up from 48%.

**Conclusion**

Ensuring safe injection practices in the CBCHS will require a multifaceted approach that focuses on surveillance, enforcement, and continuing education. Aseptic technique needs serious improvement through reminders, training, and supervision. We recommend the use of WHO and Center for Disease Control (CDC) injection safety videos and guidelines.

## A15 Introduction of multimodal hand hygiene improvement strategy in 4 hospitals in Sierra Leone

### Buyiswa Lizzie Sithole^1^, Boniface Hakizimana^1^, Christiana Kallon^2^

#### ^1^World Health Organization, Freetown, Sierra Leone; ^2^Ministry of Health and Child Welfare, Freetown, Sierra Leone

##### **Correspondence:** Buyiswa Lizzie Sithole (buyih4life@gmail.com)

**Background**

Hand hygiene is the key measure in preventing Hospital Acquired Infections and addressing the burdens. IPC practices in Sierra Leone were not coordinated. During the Ebola response period hand hygiene played an integral part of the response phase. As the country transitioned from Ebola to Health, there was need to strengthen IPC practices, especially in the area of Hand Hygiene amongst healthcare workers as this was associated with Ebola transmission-reducing practices and not basic infection prevention and control component. Sierra Leone is among the countries pledged with WHO Hand Hygiene Campaign to improve patient safety by reducing healthcare-associated infection. There is a need to scale up hand hygiene practices in health care facilities as the country is recovering from EVD outbreak.

**Methods**

Four regional Hospitals in the country were identified to start the implementation of multimodal hand hygiene improvement strategy; these are Connaught, Bo, Kenema and Makeni Government hospitals. The 4 hospitals serve as baseline data and then the assessments will be cascaded to the other health facilities. A team from WHO IPC Consultants and MOHS IPC officers carried out this self-assessment in 3 hospitals with Kenema hospital still to be visited. A hand hygiene audit tool (WHO Tool) was used to collect data in 3 hospitals; this tool involved the direct observation of hand hygiene practices among health care providers. The second tool used was the hand hygiene self-assessment framework tool (WHO Tool) which determines hand hygiene level at a particular healthcare facility. Observation of healthcare workers in various wards was observed over an hour and data entered on the tool and percentages derived.

**Results**

Hand hygiene compliance was below the standard in the three hospitals. As shown by the baseline survey, hand hygiene levels were: Inadequate with score of 87.5/500 (17.5%) at Connaught Hospital, Basic with score of 202.5/500 (40.5%) at Bo Hospital and Basic with score of 197.5/500 (39.5%) at Makeni Hospital. For direct observation of hand hygiene practices that is, identifying opportunities versus the actual actions based on 5 moments for hand hygiene, Connaught scored 46.9% (30/64), Bo 40.2% (53/132) and Makeni 18.7% (28/150).

**Conclusion**

Healthcare workers believe hand hygiene is Ebola containment measure and had no interest to maintain it since Ebola was over. There is need to set realistic targets for hand hygiene compliance and to start local production of alcohol-based hand rub to ensure it is readily accessible at the point of care.

## A16 The use of different screening methods to determine Isoniazid Preventative Therapy (IPT) eligibility among HIV infected patients at a referral hospital in Western Kenya, 2012

### Barbara Burmen (drburmen@gmail.com)

#### Kenya Medical Research Institute Center for Global Health Research, Nairobi, Kenya

**Introduction**

IPT is recommended for HIV-infected patients to eliminate LTBI. We have examined newly-diagnosed HIV-infected patients’ eligibility for IPT the using different methods at a Kenyan County hospital in a high-HIV high-TB burden region.

**Methods**

We reviewed data from a TB intensified case finding study for newly-enrolled HIV-infected patients at Siaya County Hospital in 2012 to determine IPT eligibility. The current guidelines recommend IPT initiation for patients with a negative symptom screen and patients positive symptom screen only after excluding TB. Additionally, study requirements stated that only patients with TST readings greater than 5 mm in diameter, (without TB), were eligible for IPT. Patients’ were asymptomatic if they answered no to all questions and symptomatic if they answered yes to any question on the Kenyan Ministry of Health (MOH) TB screening algorithm. Hospital-based investigations included chest x-ray and sputum ZN microscopy. Reference laboratory tests included sputum ZN and FM microscopy, culture for MTB and Gene Xpert, and stool ZN microscopy and culture for MTB.

**Results**

Of 68 patients enrolled patients, majority were female (79%) and 26 (38%) were symptomatic. The median age of all patients was 27 years (IQR 23-32.5). Twenty patients were diagnosed with TB on hospital-based screening; 14 (54%) among symptomatic and 6 (14%) among asymptomatic patients. A further 4 patients were diagnosed with TB at the reference laboratory; 2 (8%) among symptomatic and 2 (5%) among asymptomatic patients. In total, 24 (35%) patients were diagnosed with TB; 16 (62%) among symptomatic and 8 (19%) among asymptomatic patients. TST was placed and read for 6 (60%) symptomatic and 25 (74%) asymptomatic patients not diagnosed with TB; 2 symptomatic and 7 asymptomatic patients were eligible for IPT.

**Conclusion**

Intense investigations ensured appropriate patients were eligible for IPT initiation. The availability of rapid and accurate diagnostic tests would further facilitate this.

## A17 Improvement of biosafety through innovation practices at Lodwar County and Referral Hospital Laboratory, Kenya

### James Marcomic Maragia (mjmaragia@gmail.com)

#### Lodwar county and referral hospital, Lodwar, Kenya

**Background**

Biosafety has been quite challenging at Lodwar County and Referral Hospital Laboratory. This was catapulted by staff incompetence in biosafety skills in the entire Turkana County. To fulfill the requirements of section twelve (facility and biosafety) of the SLIPTA checklist, Management Sciences for health intervened to train laboratory staffs to bridge this knowledge-gap. The laboratory staffs comprehended the biosafety principles which they applied to innovate approaches for improvising the locally available materials. The aim of this paper is to describe the biosafety improvements in this resource-constrained institution following skills gained at the biosafety training.

**Methods**

Internal baseline audit was conducted using the SLIPTA checklist section twelve (Facility and Biosafety). The identified non-conformities were addressed by involving the hospital management, clinicians, laboratory staff, and maintenance staff—towards the mission of sustainable biosafety culture. Action plans were developed with clear timelines and persons responsible. A stepwise roadmap was also established in the order of priority.

**Results**

Within a year, ten out of twelve Laboratory staffs had been trained on biosafety, one master trainer in biosafety and SLMTA TOT, three internal auditors, appointment of Biosafety officer, development of biosafety manual and biosafety-related SOPs, improvised eye wash station, major signage posted and enforced, designated fire assembly point, all the staffs vaccinated, strategically placed colour-coded bins with respective bin-liners and waste segregation job aid, improvised chemical and biological spill kit, improvised first Aid kit, fire action plan and PEP protocol posted and enforced, improvised accident/incident book, chemical segregation, consistent proper use of PPE, procurement of class II biosafety cabinet, provision of clean water dispenser, environmental temperature reduced from 37oc to 20oc through installation of five functional air-conditioners, improvising hand washing sinks placing them strategically within and outside the lab, trunking all the hanging cables, presence of sand bucket and serviced fire extinguisher, safety audits done and documented, conduct of safety CMEs monthly and effective management advocacy was evidenced through mass laboratory renovations.

**Conclusion**

The was massive evidence of improved biosafety practices, tremendous positive changes have taken place due to hospital management buy-in and laboratory staff teamwork and commitment. The knowledge and skills gained through biosafety trainings can change the laboratory through innovations using the locally available resources.

## A18 Rare fungal species isolated from the oral mucosa of Libyan diabetic patients

### Mustafa Esmaio, Pedro Abrantes, Charlene Africa

#### University of Western Cape, Cape Town, South Africa

##### **Correspondence:** Mustafa Esmaio (esmaio79@gmail.com)

**Background and objectives**

Recent studies have demonstrated an increasing resistance of Candida infections to antifungals routinely used to treat candidiasis in HIV patients and in patients with diabetes mellitus (DM) and this has been attributed to the emerging resistance of different species as well as the more frequent isolation and detection of non-Candida species such as *Cryptococcus humicola, Saprochaete capitata*, and *Kloeckera apis/apiculata*. This study aimed to establish the prevalence and fluconazole resistance profiles of yeasts other than Candida, which may be found colonizing the oral mucosa of Libyan patients with DM.

**Methods**

Fungal species were isolated from the oral cavity of DM-positive patients attending a diabetes clinic in Misrata Diabetes Centre in Libya. This study included patients with DM aged between 35 and 95 years and excluded subjects who had antifungal therapy within two weeks of sample collection. The identification and characterization of the isolated species were confirmed using selective and chromogenic media, API ID 32C biochemical testing and antimicrobial susceptibility testing using disk diffusion. The study complied with the Declaration of Helsinki (2013).

**Results**

Two hundred isolates representing fifteen fungal species were identified from the oral mucosa of 173 patients, with 28.4% demonstrating resistance to fluconazole. Although Candida species predominated, other rare fungal species were also isolated such as *Cryptococcus humicola, Saprochaete capitata, Kloeckera apis/apiculata* and *Candida sake*, all of which have been associated with increased mortality in immunocompromised patients. Fluconazole resistance was observed in *Cryptococcus humicola* isolates*, Saprochaete capitata* and *Candida sake* and *Kloeckera apis/apiculata*.

**Conclusion**

Improved identification and characterisation systems allow for the identification of rare fungal species in resistant infections. This study emphasises the importance of accurate species identification and antifungal susceptibility surveillance in patients with underlying chronic diseases such as DM who may be at an increased risk of developing rare and resistant fungal infections associated with mortality due to systemic spread.

## A19 Compliance with infection prevention and control practices in six pilot public hospitals in Mozambique

### Rafael Joaquim (tarcyel@yahoo.com.br)

#### Maputo Central Hospital, Maputo, Mozambique

**Background and objectives**

Infection Prevention and Control (IPC) practices are the most important actions for reducing hospital-acquired infections (HAI), costs with hospitalization and mortality of patients worldwide, further exacerbated by the of antibiotic resistance in microorganisms associated with HAI. The Ministry of Health of Mozambique (MoH) decided in 2004 to implement an IPC programme in all health services with support of the Center for Disease Control (CDC) and International non-profit health organization affiliated with the Johns Hopkins University (JHPIEGO). The implementation started in six public hospitals around the country. The aim of this study was to assess the impact of implementation of infection prevention and control programmes in six public hospitals of Mozambique.

**Methods**

We assessed all six (N = 06) hospitals from 2004 to 2013. Data on measurable indicators was collected using a standardized checklist based in standards of IPC for assessing the organization of the program, IPC activities and availability of resources for IPC implementation. The compliance was assessed by single direct observation of procedures, interview with professionals and review of documents. The rate of each hospital was analyzed and compared with others hospitals, coefficient of variation (CV) was used to compare the baseline results and last assessment, 10 years after the start of the implementation.

**Results**

Baseline assessment of the six hospitals in 2004 ranged from 12 to 36%. After 10 years, the assessment conducted until 2013 revealed a average compliance among hospitals ranging from 62 to 86%. The baseline assessment showed more variability between hospitals (CV = 37) than last (2013) assessments (CV = 13). During this period the XaiXai Provincial Hospital achieved more than 80% in all indicators and received official recognition by the MoH.

**Conclusion**

In all hospital where the IPC program was instituted, MoH provided training and resources but the improvement is variable among hospitals. The main gaps are the infrastructure, resource and information based in IPC evidence. It is necessary to implement surveillance program and to improve and produce the IPC documents

## A20 Genetic diversity in drug resistant clinical isolates of *Mycobacterium tuberculosis* circulating within Ndola district; a high HIV prevalence district of Zambia

### Namaunga K Chisompola^1, 2^, Elizabeth M Streicher^1^, Rob M Warren^1^, Samantha L Sampson^1^

#### ^1^Faculty of Medicine and Health Sciences, Department of Biomedical Sciences, Division of Molecular Biology and Human Genetics, Stellenbosch University, Cape Town, South Africa; ^2^School of Medicine, Department of Basic Sciences, Copperbelt University, Ndola, Zambia

##### **Correspondence:** Namaunga K Chisompola (unga_k@yahoo.co.uk)

**Background and objectives**

Tuberculosis (TB) remains a major public health concern globally with a third of the worlds’ population being infected with the causative agent *Mycobacterium tuberculosis*. Progress in efforts to curb the disease is hampered by factors including the emergence of drug resistant TB strains and the HIV epidemic. In 2014, WHO reported that 1.1 million people living with HIV developed TB and 80% of these cases were reported in Africa. In Zambia, over 42, 000 TB cases were reported of which 61% were co-infected with HIV. The burden of MDR-TB in Zambia is estimated to be 8.7% for retreatment cases and 0.3% for new cases while the overall prevalence of all forms of resistance is 9.7%. The molecular epidemiology of drug resistant TB cases in Zambia is largely unknown; with only two published studies on drug susceptible TB. This research aims to address the gap in knowledge. The objectives of this research were to characterise the genetics and epidemiology of drug resistant clinical isolates of *M. tuberculosis* circulating within Ndola district. To understand the mechanisms driving drug resistant TB in Zambia, in terms of acquisition verses transmission.

**Methods**

The study involves prospective genetic characterisation of all forms of drug resistant *M. tuberculosis* clinical isolates circulating within Ndola district, Zambia, from October 2015 to October 2017. Clinical samples and data for all confirmed drug resistant TB isolates are being collected from TB diagnostic clinics, the MDR-TB ward at Ndola central hospital and Tropical diseases research centre TB reference laboratory in Ndola. Molecular characterisation is being performed at Stellenbosch University’s Molecular Biology and Human Genetics laboratory using internationally standardized techniques of Spoligotyping, IS*6110* DNA fingerprinting and whole genome sequencing.

**Results**

Preliminary findings from Spoligotyping for 52 isolates shows genetic diversity of drug resistant strains in Ndola district. Preliminary IS*6110-*RFLP results show there is some recent transmission. From the 52 isolates analysed, 3 belonged to the Beijing genotype, 4 belonged to CAS, twenty-two belonged to the LAM genotype, eleven belonged to T and 6 belonged to the X family. Six genotypes did not belong to any genotype found on the SITVIT database.

**Conclusion**

Preliminary findings show a higher than expected genotype diversity of drug resistant clinical isolates of *M. tuberculosis* circulating within Ndola district of Zambia. This suggests that acquisition is at least partially driving the MDR-TB burden in Zambia. Further, preliminary IS*6110-*RFLP results suggest that there is some recent transmission of drug resistant TB, although further samples will be needed to confirm these findings. Knowledge of the clinical isolates of drug resistant *M. tuberculosis* circulating within a given population will be beneficial in advising policy makers on infection control measures.

## A21 Chlorine inactivation of *Salmonella* Spp. recovered from selected wastewater treatment plants in Eastern Cape, South Africa

### Mojisola Christiana Owoseni, Anthony Okoh

#### University of Fort Hare, Alice, South Africa

##### **Correspondence:** Mojisola Christiana Owoseni (moji.owoseni@gmail.com)

**Background and objectives**

Water quality has been a topical issue in public health due to concerns emanating from discharge of inadequately treated sewage into water bodies. A disinfectant of choice for wastewater treatment is chlorine due to its potent oxidizing capacity in destroying microbial nucleic acid and cell membrane. Permissible limits for effluent discharge into water bodies stipulate < 1000 faecal coliform/100 mL and special standard of no risk of zero faecal coliform/100 mL. However, increasing chlorine resistance in microbial pathogens such as *Salmonella spp* has rendered chlorine inefficacious in water treatment processes. Therefore, this study investigated the chlorine inactivation efficiencies of *Salmonella spp* recovered from selected wastewater treatment plants in the Eastern Cape Province, South Africa.

**Methods**

Non-chlorinated, secondary effluents were collected from two wastewater treatment plants in the Eastern Cape Province, South Africa and processed following standard procedures (DWAF, 1996; APHA, 1999). The chlorine disinfection assay was performed for twenty Salmonella isolates in a laboratory scale study to determine free chlorine residuals and bacterial survival at the recommended chlorine dose of 0.5 mg/L. Lethal dose of chlorine and inactivation kinetics were further examined for three resistant *Salmonella* isolates at higher chlorine dosages.

**Results**

At free chlorine dose of 0.5 mg/L, residual chlorine concentrations ranged between 0.03 mg/L to 0.42 mg/L and bacterial survival ranged between 2.0 × 102 CFU/mL to 1.39 × 103 CFU/mL (99.999% inactivation) for the twenty *Salmonella* isolates. Higher chlorine concentrations of 0.75 mg/L to 1.0 mg/L markedly reduced *Salmonella* viability from 1.3 × 108 CFU/mL to a range of 0 to 3.0 × 101 CFU/mL with 0.75 mg/L free chlorine showing the highest efficiency in bacterial sterilization. Inactivation kinetics at the lethal doses showed progressive decline in bacterial survival over time and complete sterilization of *Salmonella* spp after 30 min exposure.

**Conclusion**

Chlorine dose of 0.75 mg/L to 1.0 mg/L obtained in this study was effective in bacterial reduction over time and could serve as an alternative disinfectant regimen that could facilitate the control of microbial pathogens in wastewater treatment plants in the Eastern Cape Province, South Africa. This study provides promising data for water quality in reducing the health risks associated with chlorine resistant bacterial pathogens. Future research prospects will focus on potential cytotoxic effect of higher chlorine doses on public health and the environment and this will merge laboratory study with field experiments.

Keywords: Chlorine resistance, lethal dose, inactivation kinetics, Salmonella.

## A22 Assessment of water, sanitation and hygiene (WASH), and infection, prevention and control conditions in six healthcare facilities in Western Uganda

### Habib Yakubu^1^, Katharine Robb^1^, Constance Bwire^2^, Richard Mugambe^3^, James Michiel^1^, Joanne McGriff^1^, Christine Moe^1^

#### ^1^Emory University, Rollins School of Public Health, Center for Global Safe Water, Sanitation and Hygiene, Atlanta, GA, USA; ^2^Care International in Uganda, Kampala, Uganda; ^3^Makerere University School of Public Health, Department of Disease Control and Environmental Health, Kampala, Uganda

##### **Correspondence:** Habib Yakubu (hyakubu@emory.edu)

**Background**

There is some evidence of increased maternal and neonatal mortality and healthcare acquired infections due to inadequate water, sanitation and hygiene (WASH) conditions in low and middle income countries. We developed an assessment tool to better characterize and provide evidence on the status of WASH conditions in low and middle income countries. This is to advocate and inform action for improved WASH facilities for an effective and efficient infection, prevention and control programs in low income healthcare facilities.

**Methodology**

In October 2015, the WASH condition assessment tool was deployed in six healthcare facilities in Western Uganda who had been selected but yet to receive an onsite ultra-membrane water filtration system to treat their water. The WASH conditions assessment tool consist of 1) a survey with each hospital director to understand the status of WASH conditions within a health facility, 2) an observation checklist of infrastructure, conditions and resource of toilets and key wards namely pediatrics, maternity, inpatient, outpatient and surgical of each health facility. This is to evaluate the adequacy of WASH facilities for infection, prevention and control program effectiveness. 3) Water quality sampling and analysis of the key wards to ascertain the quality of water of each hospital. The data collected was analyzed in SAS.

**Results**

Results show that none of the healthcare facilities met WHO guidelines for drinking safe water. *Escherichia coli* results from all wards ranged from 488 MPN per 100 ml to <1MPN per 100 ml. 66% of the hospitals reported proper disposal of infectious waste and 83% of the 29 wards observed had an environmental disinfectant. Only 22% of the hospitals were observed to have soap and water near their toilets. All the hospitals reported that they provide soap for their staff, however only 56% reported to provide soap to their patients.

**Conclusion**

Reduction of healthcare acquired infection and maternal and neonatal mortality due to inadequate WASH conditions requires an understanding of the status of challenges faced by healthcare facilities in low and middle income countries. The WASH conditions assessment tool results can be used to identify priority areas for improvement in infection, prevention and control programs as well as identify healthcare facilities to target for water, sanitation and hygiene intervention. It can also be used to compare conditions and track progress within and across regions to understand localized or widespread problems.

*Oral presentation prize: Bronze ICAN medal*

## A23 Hand hygiene quality improvement project at Gertrude's Children's Hospital, Nairobi, Kenya

### Jane Ngivu (jngivu@yahoo.com)

#### Infection Control Coordinator, Gertrude's Children's Hospital, Nairobi, Kenya

**Background and objectives**

Hand hygiene is the action of hand cleansing and is the single most effective way of preventing the spread of infections. Transmission of healthcare associated infections (HAIs) from one patient to another occurs mainly through contaminated hands of healthcare workers and despite this knowledge compliance has remained low. This study aimed to improve hand hygiene compliance by addressing the causes for noncompliance and consequently eliminate healthcare associated infections.

**Methods**

This study was conducted at the Inpatient department at Gertrude’s Children’s Hospital (GCH) in Nairobi Kenya. GCH attends to children from birth to 21 years of age, both at the Inpatient, Outpatient and specialized areas. The study was carried out from February to July 2015. A baseline survey was done using the World Health Organization (WHO) hand hygiene observation tool and compliance was at 63%. Baseline HAIs were 1.9/1000 bed days and root cause analysis done on all revealed noncompliance to hand hygiene as one of the main causes. Causes for poor compliance were sought for from an earlier study on hand hygiene knowledge, attitude and practices. Audit on hand hygiene facilities, sanitizers and soaps were also done as part of the baseline. Inadequate access to hand wash sinks or hand hygiene sanitizers and unfriendly soaps were found to be the main causes for the poor compliance. Changes implemented were; staff training on hand hygiene with special emphasis on the How, When and Why, hand hygiene sanitizers were installed in several areas which were lacking and hand wash soap was changed to one which was more user friendly. Routine observational monitoring of hand hygiene compliance by trained observers was done. Data was analyzed using Ms Excel and individualized feedback given to staff and their respective managers throughout the study period.

**Results**

HAIs reduced from a baseline of 1.9/1000 to zero by end of the study period. Irritability from hand wash soap was eliminated and hand hygiene compliance improved by 17%; from 63% to 80%.

**Conclusion**

Hand hygiene compliance can be improved if the reasons for noncompliance are identified and addressed. There is need to provide adequate access to hand wash sinks and hand hygiene sanitizers. User-friendly products should be utilized to enhance compliance and train healthcare workers on hand hygiene; the How, When and Why to foster their compliance. More research is needed to find out other causes for noncompliance since the compliance is still not at 100%.

## A24 Indiscriminate use of antimicrobials: A call for the implementation of antimicrobial stewardship programme

### Olanrewaju Jimoh^1^, Oluwafemi T. Ige^2^, Zainab L Tanko^1^, Abdulmumin K. Mohammed^1^, Victoria Aganabor^3^, Busayo O. Olayinka^4^, Abdulrasul Ibrahim^1^, Joy O. Daniel^3^, Adebola T. Olayinka^1^

#### ^1^Department of Medical Microbiology, Ahmadu Bello University /Teaching Hospital, Zaria, Kaduna State, Nigeria; ^2^Department of Medical Microbiology, Kaduna State University, Kaduna, Nigeria; ^3^Department of Nursing services, Ahmadu Bello University /Teaching Hospital, Zaria, Kaduna State, Nigeria; ^4^Department of Pharmaceutics and Pharmaceutical Microbiology, Ahmadu Bello University, Zaria, Kenya

##### **Correspondence:** T. Olayinka (debolaola@yahoo.com)

**Background**

Antimicrobials are non-replaceable in the treatment of severe bacterial infections. It is therefore important that they should be preserved and used judiciously. In recent times a lot of focus has been on the increase in antimicrobial resistance and type of resistance. Selective pressure has been a main contributor to antimicrobial resistance. In Nigeria there is no restriction on the prescription and sale of antimicrobials both within the community and in the hospitals. This study was to assess the antimicrobial prescription pattern in a tertiary hospital in North western Nigeria.

**Methods**

A point prevalence survey was carried out in all wards of the hospital in June 2015. All in-patients receiving an antimicrobial agent on the day of the survey were included. Data was obtained using a structured questionnaire and abstraction from medical records. Information obtained included demographic data, antimicrobial agents used, indication for treatment, microbiological data and stop/review dates. Data was analyzed using SPSS version 20.0 and descriptive statistics documented.

**Result**

Twenty-three wards with a total number of 318 in-patients were recruited for the study. Male: Female ratio was1.2:1, participants median age in adults (35 years, range 2-75 yrs), in children (9 months, range 2-21months) and in neonates (8 days, range 1-30days). Of all patients on admission, 210/318 were on antimicrobials giving an antimicrobial prevalence rate (AMR) of 66%. Ward AMR ranged from 7.7% (Psychiatry Ward) to 95.5% (Emergency Paediatrics ward). A total of 331 antimicrobials were prescribed. The top indications for use were surgical prophylaxis 146/331 (44.1%), community-acquired infection 101/331 (30.1%), medical prophylaxis 45/331 (13.6%) and unknown indication 25/331 (7.6%). The three commonly prescribed class of antimicrobials were cephalosporins 95 (28.7%) of which ceftriaxone was the highest 61/95 (64.2%); imidazoles 70 (21.1%) of which only metronidazole (100%) was prescribed, followed by the quinolones, 50 (15.1%) with ciprofloxacin 44/50 (88.0%) being the commonest of the prescribed quinolones. Majority 327 (98.8%) of the prescriptions were based on empirical antimicrobial treatment and 285 (86.1%) were open prescriptions with no review/stop date documented.

**Conclusion**

This survey was the first of its kind on antimicrobial prescription in this hospital. There is a high prevalence of antimicrobial use in our facility and major indication for use was as prophylaxis. Only few cases had targeted treatment and many prescriptions were left open. These findings demonstrate the need to have an antimicrobial stewardship programme in place with proper implementation and buy-in of prescribers to ensure rational use of antimicrobials.

## A25 Infection control is an essential part of antimicrobial stewardship in an ICU

### Joan Rout^1^, Petra Brysiewicz^2^

#### ^1^Hillcrest Private Hospital, Durban, South Africa; ^2^School of Nursing and Public Health College of Health Sciences University of KwaZulu-Natal, Durban, South Africa

##### **Correspondence:** Joan Rout (joanrout@worldonline.co.za)

**Background and objectives**

The care of the critically ill patient across the world has become more challenging with increasingly resistant pathogens resulting in difficult to treat infections. Severe infections increase the length of time spent in an intensive care unit (ICU), increases morbidity and mortality, and increases healthcare costs. Infection prevention and control measures are an essential part of antimicrobial stewardship in preventing emergent resistant pathogens and hospital-acquired infections. Hospitals in South Africa are facing the growing challenge of micro-organisms resistant to routine antibiotic therapy due to ineffective infection prevention and control and overuse of antibiotics in both medical and veterinary fields. The objective of this study was to identify the various aspects of the role played by the Critical Care Nurse as a member of the antimicrobial stewardship team in a private healthcare facility in South Africa.

**Methods**

A qualitative approach using content analysis was used in this study. Purposive sampling identified participants from an ICU multidisciplinary antimicrobial stewardship team in a private hospital. Semi-structured interviews were conducted with fifteen participants; ICU clinical nurses, nursing management, surgeons, anaesthetists, physicians, microbiologists and pharmacists. Findings from the participants were divided into two groups; nursing and non-nursing participants.

**Results**

Perspectives of the various members of the multidisciplinary antimicrobial stewardship team identified a large part of the clinical role of the ICU nurse as monitoring for infection and infection control. Nursing participants were concerned about poor compliance with universal infection prevention and control measures within the ICU by nurses and doctors. Non-nursing participants suggested that there was inadequate communication from the infection prevention and control coordinator. Other important aspects of the role of the ICU nurse were found to be organizational, advocatory and collaborative.

**Conclusion**

This study has established that all disciplines of healthcare workers in ICU understand that the care of the critically ill patient can be complicated by difficult infections and ineffective antibiotics. The value of the ICU nurse in antimicrobial stewardship has been identified as; organizing shift leader antimicrobial stewardship rounds and antimicrobial stewardship meetings, diligent monitoring of the ill patient for signs of infection, documenting and communicating these findings to other members of the antimicrobial stewardship team, administering antimicrobial therapy correctly and preventing hospital-acquired infections. Further research needs to be carried out into the various aspects of the role of the ICU nurse in antimicrobial stewardship, optimal staffing of ICUs and nurse/doctor collaboration.

## A26 SSI-Bundle: making a difference by implementing two evidence-based elements for all Caesarean cases

### Yolanda Van Zyl; Shereen Arontjies

#### Paarl Hospital, Western Cape, Paarl, South Africa

**Background and objectives**

Paarl Hospital (301 beds) caters to the general and emergency care needs of a population of over 600 000 encompassing a predominantly semi-rural region in the Southern tip of South Africa. TB and HIV still remains the biggest challenge when it comes to strengthening IPC programmes. The Surgical Site Infection (SSI) -Bundle^1^ was added to the Annual Operating plan (2014/2015) for all public hospitals in the Western Cape Province. The hospital was familiar with the use of infection prevention bundles but had never implemented the SSI-Bundle.

**Methods**

Bundle piloted in all Caesarean section patients (20% of +/-450 deliveries a month). Days between infections were average 20 days. Goal was set to reach >150 days between infections using two bundle elements: the use of clippers for hair removal and administering antibiotic prophylaxis <60 min prior to incision. At the time prophylaxis was administered in theatre by the anaesthetist after the umbilical cord was clamped. This practice was changed: antibiotic prophylaxis administered in ward at the same time as the pre-medication. Two additional elements were added: Showering with chlorhexidine soap on the morning of surgery and wearing a disposable cap to cover hair. We combined a SSI-bundle checklist with the existing CAUTI-insertion checklist already in use. Real time audits using a checklist was done to determine bundle compliance and outcomes

**Results**

Real time audits revealed that the median compliance rate was 90% for administering antibiotic prophylaxis <60 min prior to incision. Days between infections increased from a maximum of 20 days to a record of 173 days on 11/08/2015 after which there had been 11 infections at the time writing, 5/09/2016.

**Conclusion**

In low resource countries, the option of ‘No hair removal” can be implemented rather than procuring the clippers. Focus on making small changes and still get significant results. Only two elements of the bundle was implemented. The biggest reward is that less patients had to suffer the trauma of an infected wound at the time they had brought a new baby into their families. Although the SSI-bundle elements is evidence based it will be interesting to see the difference in outcomes between using clippers and not removing hair.

**Fig. 1 (abstract A26).**
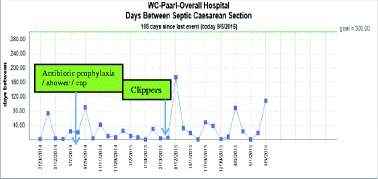
See text for description

